# Chlamydia Outer Protein (Cop) B from *Chlamydia pneumoniae* possesses characteristic features of a type III secretion (T3S) translocator protein

**DOI:** 10.1186/s12866-015-0498-1

**Published:** 2015-08-14

**Authors:** David C. Bulir, Daniel A. Waltho, Christopher B. Stone, Steven Liang, Christopher K. W. Chiang, Kenneth A. Mwawasi, Jordan C. Nelson, Steven W. Zhang, Samantha P. Mihalco, Zachariah C. Scinocca, James B. Mahony

**Affiliations:** M. G. DeGroote Institute for Infectious Disease Research, Faculty of Health Sciences and Department of Pathology and Molecular Medicine, McMaster University, Hamilton, ON Canada; Father Sean O’Sullivan Research Centre, St. Joseph’s Healthcare, Hamilton, ON Canada; Regional Virology Laboratory, St. Joseph’s Healthcare, 50 Charlton Ave. E, Hamilton, ON L8N 4A6 Canada

## Abstract

**Background:**

Chlamydia *spp*. are believed to use a conserved virulence factor called type III secretion (T3S) to facilitate the delivery of effector proteins from the bacterial pathogen to the host cell. Important early effector proteins of the type III secretion system (T3SS) are a class of proteins called the translocators. The translocator proteins insert into the host cell membrane to form a pore, allowing the injectisome to dock onto the host cell to facilitate translocation of effectors. CopB is a predicted hydrophobic translocator protein within the chlamydial T3SS.

**Results:**

In this study, we identified a novel interaction between the hydrophobic translocator, CopB, and the putative filament protein, CdsF. Furthermore, we identified a conserved PxLxxP motif in CopB (amino acid residues 166–171), which is required for interaction with its cognate chaperone, LcrH_1. Using a synthetic peptide derived from the chaperone binding motif of CopB, we were able to block the LcrH_1 interaction with either CopB or CopD; this CopB peptide was capable of inhibiting *C. pneumoniae* infection of HeLa cells at micromolar concentrations. An antibody raised against the N-terminus of CopB was able to inhibit *C. pneumoniae* infection of HeLa cells.

**Conclusion:**

The inhibition of the LcrH_1:CopB interaction with a cognate peptide and subsequent inhibition of host cell infection provides strong evidence that T3S is an essential virulence factor for chlamydial infection and pathogenesis. Together, these results support that CopB plays the role of a hydrophobic translocator.

## Background

*Chlamydia* infections represent a significant disease burden worldwide. *C. trachomatis* infection can lead to pelvic inflammatory disease (PID), salpingitis, and infertility in women and epididymitis and infertility among men [1]. Furthermore, *Chlamydia pneumoniae* is a respiratory pathogen causing approximately 10 % of community acquired pneumonia [2]. Additionally, *C. pneumoniae* infections have been associated with asthma exacerbations, cardiovascular disease, Multiple Sclerosis, and Alzheimer’s [3–7]. Combined, *C. pneumoniae* and *C. trachomatis* represent a significant disease burden. An essential component of *Chlamydia*’s survival is creating an environmental niche that exhibits the necessary requirements for replication and survival. Type III secretion (T3S) is a complex mechanism utilized by important Gram-negative bacterial pathogens. *Salmonella*, *Shigella*, *Yersinia*, *Pseudomonas*, and *Chlamydia* all contain the highly conserved type III secretion system (T3SS) of approximately 20–30 proteins [8–11]. To manipulate their host environment, these bacteria secrete toxic effector proteins directly into their target cell. Functionally, the whole apparatus can be referred to as an injectisome; however, it consists of smaller functional components, which include the cytoplasmic C-ring, the inner and outer membrane rings, the needle complex, and needle-tip complex [8–10, 12]. Each of these components display numerous essential protein-protein interactions. Despite the identification and characterization of many putative T3S proteins, it remains unclear whether *Chlamydia* truly has a functional T3SS, and whether it plays a role in replication and survival given the absence of a robust genetic manipulation system for gene knockouts [13].

*Chlamydia spp*. undergo a unique biphasic life-cycle starting with an infectious, non-metabolically active elementary body (EB) [14–16]. Upon attachment of the EB to the host cell, there is a conformational change within the host membrane that allows for invasion of the EB into a membrane-derived vacuole termed an inclusion [16]. Once inside the inclusion, an as of  yet unknown signal triggers differentiation of the EB into a metabolically-active, non-infectious reticulate body (RB) that divides through binary fission until late in the infection cycle [16]. The infectious EB will then leave either through a packaged release mechanism, called extrusion, or through cell lysis, to repeat the infection cycle [17–19]. Throughout this process the T3SS is belived to play an essential role in pathogenicity [12].

The translocator proteins of the T3S system are believed to be critical to the survival of *Chlamydia,* by forming a pore in the host cell membrane to allow for translocation of effector proteins from the bacterial cytosol to the host cell cytoplasm [8–10]. Analysis of the chlamydial genome suggests that there may be two sets of translocator proteins, CopB and B2 and CopD and D2, both of which are located in the same operon as a predicted class II chaperone [20]. To date, there has been limited characterization of the translocator proteins from *Chlamydia spp.* Early work on the translocator proteins in *Chlamydia* indicated that both CopB and CopB2 can be secreted from *Yersinia spp.* in a T3S-dependent manner and that Scc2 co-precipitated with CopB from a *C. trachomatis* infected monolayer [21]. More recently, localization experiments have shown that CopB and CopB2, when ectopically expressed in HeLa cells, associate with the cytoplasmic and inclusion membrane, respectively [22]. Our laboratory has previously characterized the minor hydrophobic translocator (CopD) from *Chlamydia pneumoniae*. We have shown that it associates with T3S components and contains an essential PxLxxP motif for interaction with its class II chaperone, LcrH_1 [23]. Although many hypotheses can be made regarding the possible function of the translocator proteins based on orthologous T3SS translocator proteins, there is limited information on the biochemical characterization of chlamydial translocator proteins owing to the inherent difficulties of working *Chlamydia spp*.

In this report, we characterize the putative T3SS translocator protein CopB of *C. pneumonia,* explore interactions between CopB and other T3SS proteins, and characterize the chaperone binding domain of CopB. In addition, we generated a novel peptide mimetic that blocks the interaction between the translocators, CopB and CopD, and their chaperone, LcrH_1, and showed that the peptide mimetic prevents infection. We also identify a CopB epitope which is immunogenic and elicits neutralizing antibodies that block *C. pneumoniae* infection supporting an essential role for CopB in the infection of host cells.

## Methods

### Cloning

T3SS genes were cloned via PCR using genomic DNA from *C. pneumoniae* CWL029 [23]. Fragments of CopB, excluding the transmembrane domains, were cloned due to toxicity of full length CopB in *E. coli.* Using the Gateway cloning system (Invitrogen) the following genes were cloned into the pDONR201 vector with *attB*-containing primers (note: subscript denotes amino acid number): *lcrH_1, copN, copD*_*1–157*_*, cpn0803, copB*_*1–180*_*, copB*_*1–200*_*, copB*_*1–255*_*, copB*_*275–382*_*, copB*_*407–493*_*,*^*P166A*^*copB*_*1–200*_*,*^*L168A*^*copB*_*1–200*_*, and*^*P168A*^*copB*_*1–200*_. Each of the pDONR201 vectors were then used in an LR reaction to transfer the gene into expression vectors, pDEST17 (N-Terminal 6xHis-tag), pDEST15 (N-Terminal GST-tag) and pDEST-HisMBP (N-Terminal 6xHis-Maltose Binding Protein-tag). Prior to protein expression, all constructs were verified by Sanger sequencing at the MOBIX laboratory (McMaster University).

### Protein expression and purification

All constructs were transformed into *E. coli* BL21 and recombinant protein was expressed following induction with Isopropyl β-D-thiogalactopyranoside (IPTG). Protein expression and purification were performed as described by Bulir *et al.* (2014), with the following modifications [23]. Briefly, 6 L of LB containing 100 μg/mL ampicillin was inoculated with 1:100 dilution of an overnight culture and split equally into 6x 2 L flasks. The cultures were then grown at 37 °C with shaking at 250 RPM until an optical density of 0.500 at 600 nm was reached. Cultures were induced with 0.2 mM IPTG and were left incubating at room temperature, shaking at 250 RPM for 3 h.

### Glutathione-S-transferase (GST) pull-down assay

Glutathione-S-transferase pull-down assays were performed as described by Bulir et al. (2014) [23]. Briefly, GST-tagged proteins were bound to 1 mL GST beads for one hour at 4 °C on a mixing platform. GST beads were centrifuged at 3000 x *g* for 5 min to remove the supernatant and then blocked with blocking solution (5 % BSA in PBS + 0.1 % TWEEN-20) overnight at 4 °C. Blocked beads (50–100 μL) were mixed with *E. coli* lysates containing overexpressed His-tagged protein for one hour. For experiments involving the blockade of interaction between GST- and His-tagged constructs, the chemically synthesized peptide was incubated with the bait construct for 1 h at 4 °C prior to the addition of the overexpressed His-tagged *E. coli* lysate. The beads were then centrifuged at 16,000 × *g* for 10 s, the supernatant was removed, and the pellet was washed with high salt wash buffer (500 mM KCl, 20 mM Tris–HCl pH 7.0, 0.1 % Triton X-100). The washing procedure was repeated seven times to ensure complete removal of adventitiously bound protein. For GST pull-downs involving synthetically produced peptide, the peptides were used at a concentration of 500 µM. The glutathione-agarose beads were then resuspended in 75 μL of SDS-PAGE loading dye. The samples were analysed by SDS-PAGE and Western blot analysis using a mouse anti-His antibody (GenScript, New Jersey).

### Bioinformatics

Orthologous proteins to CopB were identified using BLASTP (Basic Local Alignment Search Tool Protein) and PSI-BLAST, excluding *Chlamydiaceae* family from the search. CopB was analyzed using the TMpred software tool to predict transmembrane domains, using a minimum transmembrane window of 17 and maximum of 33. Coiled-coil prediction software, COILS, was used to predict the presence of coiled-coil domains within CopB, using the MTIDK scoring matrices, and weighting for positions a & d.

### Antibody and peptide inhibition of *C. pneumoniae* infection in HeLa cells

Infection was performed as previously described by Johnson et al. [15]*.* At approximately 72 h post infection, chlamydial inclusions were stained with the Pathfinder *Chlamydia* detection reagent (BioRad) and visualized with multiple, random fields of view. Percent reduction of infection was calculated compared to a control infection, and statistical significance was calculated using a Student’s *t*-test. A polyclonal antibody raised against a 15 amino acid peptide (SGKDKTSSTTKTETC) from CopB was obtained from GenScript (New Jersey). *C. pneumoniae* was pre-incubated for 2 h at 37 °C with dilutions of affinity purified CopB antibody, control antibody (anti-GST), or pre-immunization sera. Additionally, chlamydial infection inhibition was performed using a synthetic peptide (500 uM), vehicle alone (PBS), or control peptide (anti-RSV peptide). Briefly, 5×10^5^ IFUs were incubated with the peptide or vehicle alone (PBS) for 2 h at 37 °C prior to performing a standard infection and inclusions were visualized as previously described.

## Results

### Bioinformatic analysis of Chlamydia outer protein (Cop) B

**Fig. 1:**
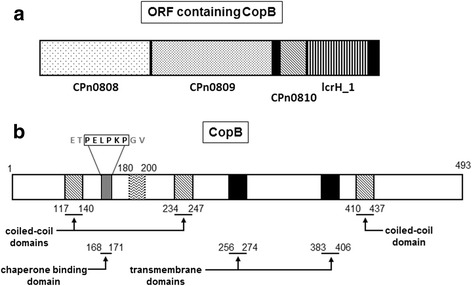
Genetic organization and topographic overview of structural prediction of CopB. Solid black regions represent transmembrane domains. (**a**) Genetic organization of the ORF containing the putative translocator, CopB and CopD, and the TPR-domain containing chaperone, LcrH_1. (**b**) Diagonal stripes represent predicted coiled-coil domain in the C-terminus of the protein. Vertical stripes depict predicted Chaperone Binding Domain (CBD) spanning amino acid residues 168–171. The hydrophobic region is shown from amino acids 180–200

Translocator proteins have a conserved function across numerous bacterial species, facilitating the translocation of effector proteins from the bacterial cytosol to the host cell cytoplasm through formation of pores within the host cell membrane. However, there is limited sequence orthology between *Chlamydia spp.* translocators and other well-characterized bacterial translocator proteins. BLAST-P analysis identified potentially orthologous sequences in the recently sequenced genome of *Bacteroides fragilis* with an expect value of 6e^−141^ and percent identity of 54 %. CopB is a 493 amino acid protein with a predicted molecular weight of 50.5 kDa. Potential transmembrane domains were identified using online prediction software, TMpred, which suggests the presence of two transmembrane domains, spanning amino acids 256–274 and 383–406, respectively, and a hydrophobic stretch of amino acids from 180 to 200. COILS software identified three potential coiled-coil domains located at amino acids 117–140, 234–347, and 410–437. Sequence analysis of the N-terminal region of CopB identified a conserved chaperone binding motif of PxLxxP at amino acids 166–171 with the sequence of PELPKP (Fig. 1). Together, these results are consistent with features characteristically found in T3S translocator proteins [22, 23].

### CopB interacts with the putative needle filament protein, CdsF

**Fig. 2:**
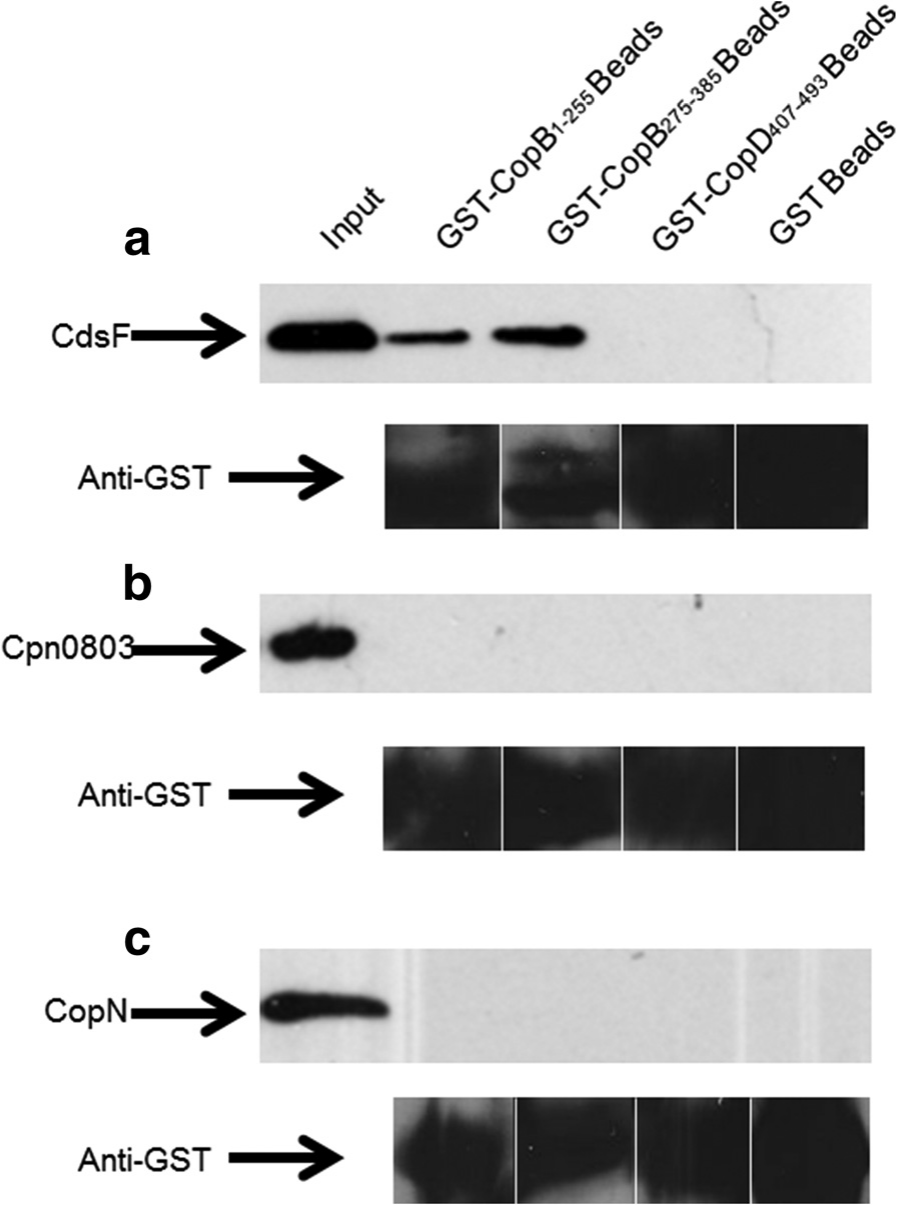
Chlamydia Outer Protein (Cop) B Interacts with T3S proteins. (**a**) GST-CopB_1–255_ or GST-CopB_275–385_ bound to glutathione-agarose beads (bait) pulled HisMBP-CdsF (prey) out of an *E. coli* lysate in the presence of a high salt wash buffer (500 mM NaCl). (**b** & **c**) Furthermore, GST-CopB fragments did not pull His-CopN or Cpn0803 out of an *E. coli* lysate in the presence of a high salt wash buffer

CopB is believed to be a T3S protein, and thus it should interact with other proteins within the T3SS [10]. Cloning fragments of CopB lacking the transmembrane domains allowed us to identify specific domains of CopB that are responsible for interactions with other type III secretion components. GST pulldowns were performed between CopB and Cpn0803, CdsF, and CopN. No interactions were observed between any fragments of CopB and Cpn0803 or CopN (Fig. 2a and b). There was a positive interaction between the N-terminal (amino acids 1–255) and middle fragment of CopB and CdsF, but not the C-terminus of CopB (Fig. 2c). These observations are consistent with a role in the T3S apparatus of *Chlamydia pneumonia*, since translocator proteins from orthologous systems have been shown to interact with the needle filament protein.

### LcrH_1 interacts within the N-terminus of CopB

**Fig. 3:**
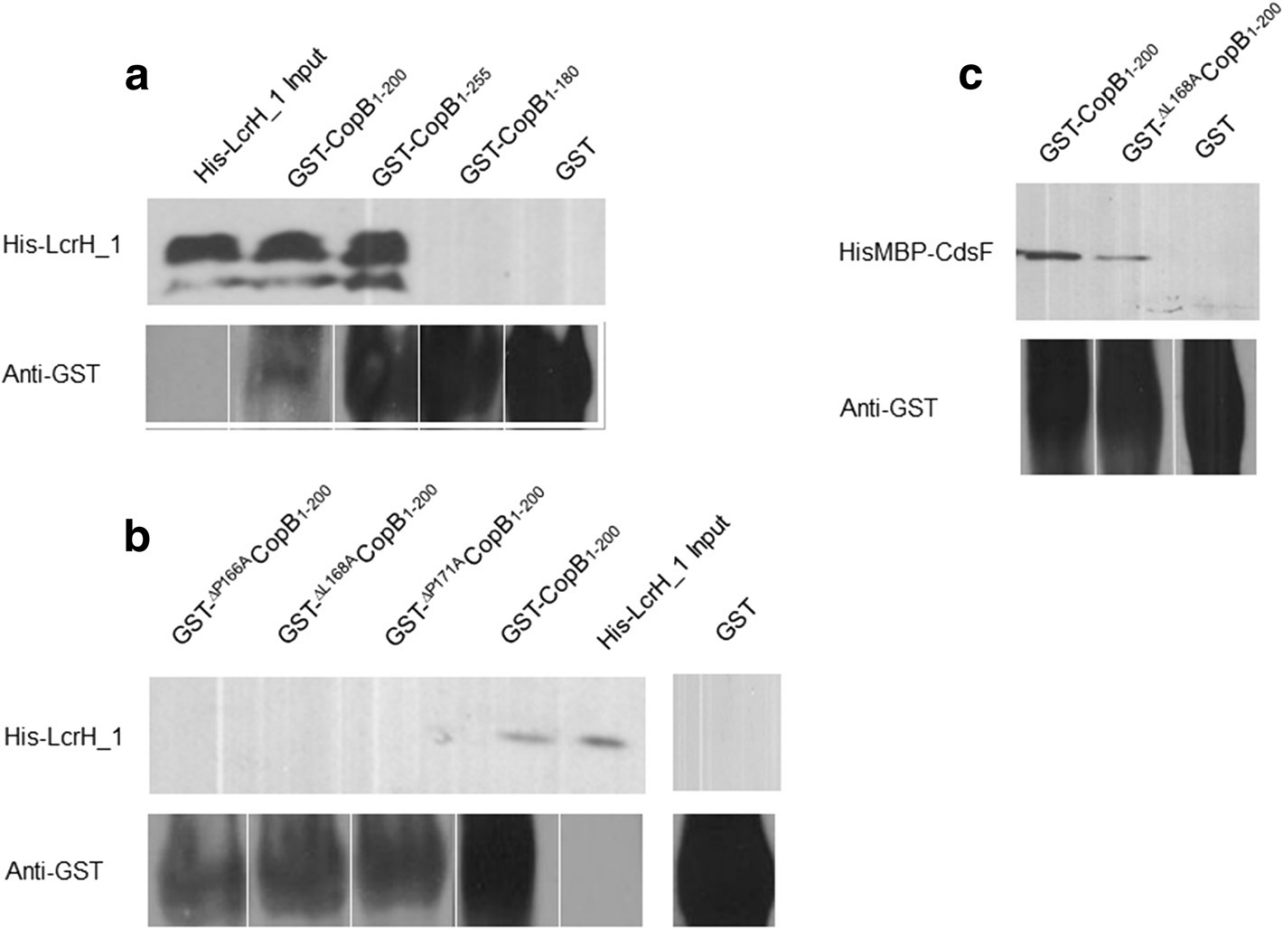
LcrH_1 (*Cpn0811)* interacts with CopB. (**a**) Recombinant LcrH_1 interacted with amino acids 1–200 of CopB. CopB mutants were created using Gblock synthesis: ^P166A^CopB_1–200_, ^L168A^CopB_1–200_, and ^P171A^CopD_1–200._ (**b** & **c**) Mutations at the conserved amino acids within the predicted chaperone binding domain disrupted the interaction between CopB_1–200_ and the chaperone LcrH_1, but not other identified interactions

Cpn0811 (LcrH_1) is a small, basic isoelectric protein located upstream in the same operon as CopB (Cpn0809) [20]. We explored the possible interaction between LcrH_1 and CopB and found that His-LcrH_1 interacts within the N-terminus of CopB (Fig. 3a). Both CopB_1–200_ and CopB_1–255_ interacted with His-LcrH_1, but CopB_1–180_ did not, suggesting the hydrophobic stretch of amino acids spanning residues 180–200 plays an important role in this interaction. Since CopB_1–200_ was the smallest truncation construct that maintained an interaction with His-LcrH_1, we examined the amino acid sequence for the presence of a conserved chaperone binding motif, PxLxxP, which begins at amino acid 166. To elucidate the importance of the conserved motif, we performed an alanine walkthrough of the conserved amino acids in the PxLxxP motif starting at amino acid 166(^P166A^CopB_1–200_, ^L168A^CopB_1–200_, ^P171A^CopB_1–200_). Mutation of the PxLxxP motif abrogated the interaction between His-LcrH_1 and CopB (Fig. 3b). To ensure that the absence of interaction was the result of the specific amino acid substitution, as opposed to gross misfolding of the mutant protein, ^L168A^CopB_1–200_ was subjected to a GST pulldown against CdsF. As expected, ^L168A^CopB_1–200_ maintained the interaction with HisMBP-CdsF (Fig. 3c), suggesting that the PxLxxP is a critical interaction domain between the chaperone and CopB.

### A CopB peptide mimetic blocks the LcrH_1 and CopB interaction

**Fig. 4:**
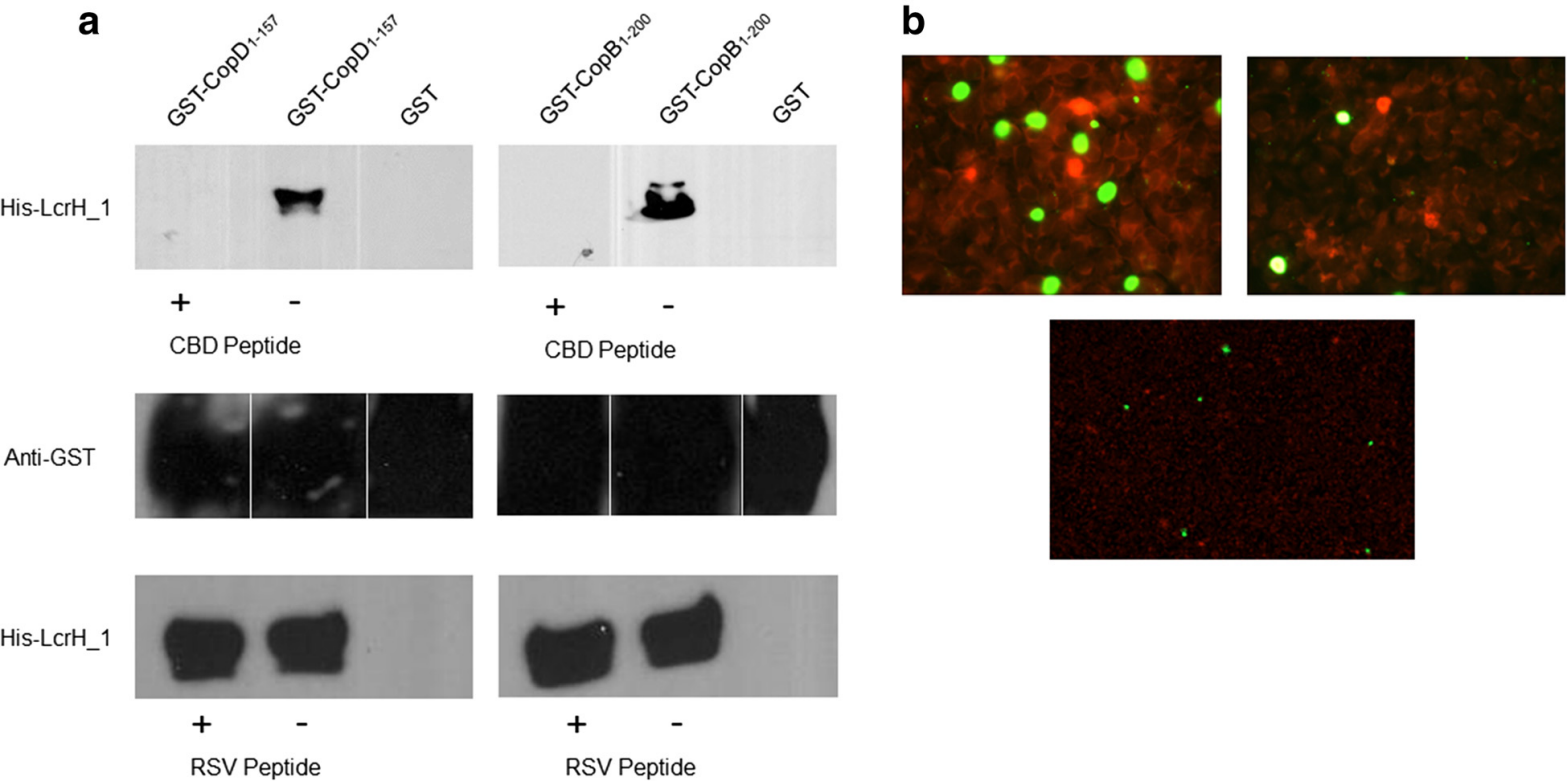
Peptide inhibition of the translocator:chaperone interaction. (**a**) Recombinant GST-CopD_1–157_ or GST-CopB_1–200_ was pre-incubated with 500 μM of the chaperone binding domain peptide mimetic (+) or vehicle alone (−). CopD_1–157_ and GST-CopB_1–200_ did not interact with its putative chaperone in the presence of the CBD peptide, but did so in the presence of a control peptide or vehicle alone. (**b**) Left image is *C. pneumoniae* incubated with vehicle alone (PBS), right image is *C. pneumoniae* incubated with 500 μM CBD Peptide, bottom image is *C. pneumonia* incubated with 50 uM anti-RSV peptide. Chlamydial inclusions are stained green, while HeLa cells are stained red by Evan’s blue counterstain

Given the necessity of the PxLxxP motif for the interaction between translocator proteins and their class II chaperones, a synthetic peptide containing the chaperone binding motif was synthesized and tested for its ability to block the interaction between LcrH_1 and both CopB and CopD. To determine whether a synthetic peptide consisting of a cell penetrating peptide sequence (YGRKKRRQRRR) and the 10 amino acids (ETPELPKPGV) encompassing the chaperone binding motif of CopB is capable of preventing the chaperone:translocator interaction, the peptide was incubated with GST-CopB_1–200_ or GST-CopD_1–157_ prior to the addition of His-LcrH_1. In the presence of the peptide, no interaction was observed between the putative translocators, CopB and CopD, and LcrH_1 under high salt conditions (Fig. 4a). To explore the hypothesis that the CopB:LcrH_1 and CopD:LcrH_1 interaction are essential for infection, we then tested the ability of the peptide to block *C. pneumoniae* infection. We pre-incubated *C. pneumoniae* with the peptide or vehicle alone and then infected host cells. The peptide inhibited infection by 90 % compared to the control infection with vehicle alone (Fig. 4b).

### Anti-CopB antibody inhibits *C. pneumoniae*

**Fig. 5:**
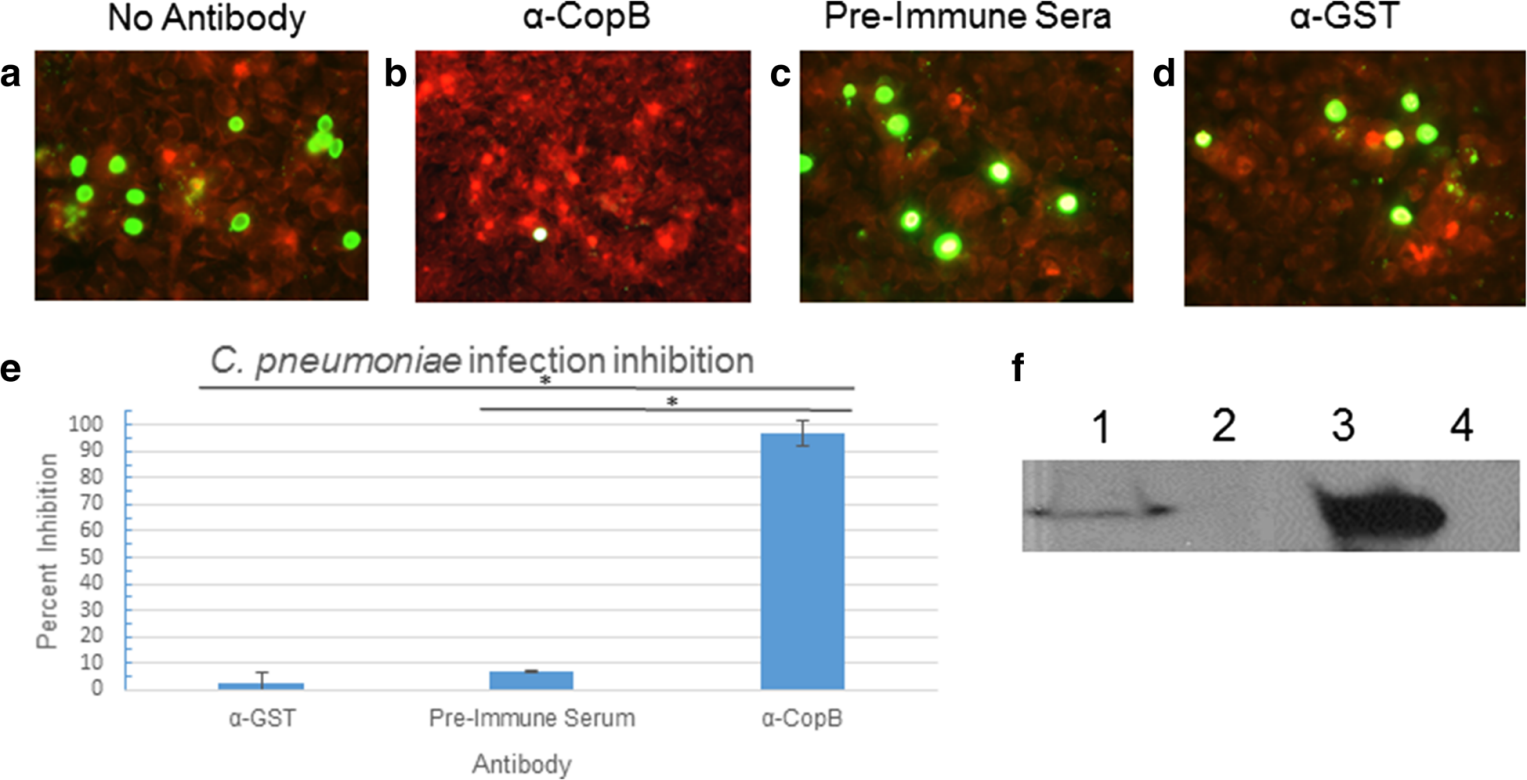
Inhibition of *Chlamydia pneumoniae* with CopB antibodies. Panels **a**-**d** show inhibition assay results performed with either no antibody (**a**), CopB antibody (**b**), pre-immune sera (**c**), or control antibody (α-GST) (**d**). Panel **e** shows the degree of inhibition by of CopB antibodies compared to control antibodies. Chlamydial inclusions are stained green, while HeLa cells are stained red by Evan’s blue counterstain. Panel **f** demonstrates reactivity of anti-CopB with (1) *C. pneumoniae* infected HeLa cell lysate, (2) uninfected HeLa cell lysate, (3) recombinant GST-CopB_1–255_ produced in *E. coli*, and (4) recombinant GST produced in *E. coli.* Experiments were performed in triplicate. Error bars represent 2 standard deviations. Images represent random fields of view. * = *P* < 0.0001

Since T3S translocators are believed to be surface exposed proteins in other T3SS, we hypothesized that antibodies to CopB would inhibit infection [24–26]*.* We generated an antibody to a peptide (15-mer) in the N-terminal region of CopB and tested its ability to inhibit *C. pneumoniae* infection. To test whether this antibody could inhibit infection, we pre-incubated *C. pneumoniae* with the polyclonal antibody for 1 h at 37 °C prior to infection. *C. pneumoniae* infection was inhibited by the CopB antibody. (Fig. 5a-d), resulting in a 98 % reduction in inclusion forming units, as compared to control antibody (Fig. 5e). Using a Western blot, polyclonal antibodies were able to detect both recombinant and native CopB (Fig. 5f). The ability of the CopB antibody to block infection suggests that CopB is surfaced exposed, and plays a critical role in the infection process.

## Discussion

Despite our increasing understanding of the T3SS in *Chlamydia spp.*, there is limited or no evidence for a direct role for the translocator proteins during infection. Our laboratory has previously characterized the putative minor hydrophobic translocator, CopD, showing that it plays an essential role during chlamydial infection [23]. In this report, we provide an initial characterization of the major hydrophobic translocator, CopB. The interaction of CopB with the filament protein CdsF suggests that it plays an essential role in T3S. As seen with other translocator proteins, the putative chaperone located immediately upstream of CopB interacted with the first N-terminal 200 amino acids of CopB. Using an alanine walkthrough of the conserved PxLxxP motif, we show that amino acids P166, L168, and P171, in addition to amino acids 180–200, are required for the interaction between CopB and its’ cognate chaperone LcrH_1. We demonstrated that a cognate CopB peptide encompassing the chaperone binding motif can block the interaction between LcrH_1 and both CopB and CopD, suggesting that the CBD is a critical binding domain. Furthermore, we show that this peptide when pre-incubated with *C. pneumoniae*, blocked infection. Together, these results strongly suggest that the PxLxxP motif is required for the translocator-chaperone interaction, and for infection. We also show that a polyclonal antibody raised against an N-terminal epitope within CopB significantly reduced infection. Collectively, these results are consistent with CopB’s role as a translocator within the C*hlamydia* T3SS.

Initial bioinformatic studies were performed to gain insight into the role of CopB in *C. pneumoniae* [21–23]. Chellas-Géry et al. identified potential hydrophobic and coiled-coil domains within CopB from *C. trachomatis* [22]. Given the moderate level of sequence identity between *C. trachomatis* and *C. pneumoniae* CopB (approximately 52 % amino acid identity), a thorough bioinformatics analysis of CopB was performed. BLASTP analysis of CopB yielded one significant result from *Bacteroides fragilis*, typically a commensal bacterium found in the gastrointestinal tract, but no matches were found for other T3S systems, suggesting that the *C. pneumoniae* T3SS may be quite unique among orthologous systems, which is in keeping with *Chlamydiae* containing an ancient T3SS. Although no orthologous sequences of the chlamydial translocator proteins were identified in the archetypal secretion systems using our bioinformatics approach, the proteins are predicted to have similar structure and function. Since CopB is likely anchored within the host-cell cytoplasm to facilitate translocation of effector proteins, we utilized TMpred online software to identify potential transmembrane regions. Our analysis identified two potential transmembrane domains spanning amino acids 256–274 and 383–406, respectively. This is consistent with other translocator proteins possessing two transmembrane domains to anchor themselves within the host cell membrane [27, 28]. Using the COILS online prediction software, we identified three potential coiled-coil domains, which may be important for mediating protein-protein or protein-membrane interactions. The N-terminus of CopB contains a conserved PxLxxP motif, followed by a sequence of hydrophobic amino acids, which is seen in other translocator proteins from *C. pneumonia,* and has been shown to be important for mediating the essential translcoator:chaperone interaction in other bacterial systems (*Shigella, Yersinia, Salmonella*) [23].

Due to the difficulty in genetically manipulating *Chlamydia* and the inherent challenges of establishing structure-function relationships for T3S proteins of obligate intracellular pathogens dependent on T3S for infection, it is difficult to ascertain the role of chlamydial T3SS proteins. We therefore explored the possible interaction between CopB and other proteins within the chlamydial T3SS. The needle filament protein, CdsF in *Chlamydia*, is believed to polymerize forming needle structure for the translocation of effector proteins. The translocator proteins are believed to be docked on the tip of the injectisome to form the needle-tip complex, prior to host cell contact. Two domains of CopB, amino acids 1–255 and 275–382, interacted with CdsF using a GST-pulldown assay. CopN, the putative plug protein, is believed to be localized to the base of the needle apparatus and prevents premature secretion of effector proteins. No apparent interaction between CopN and CopB was observed using a GST-pulldown assay. Although an interaction was observed between CopD and CopN, the lack of interaction between CopB and CopN suggests that CopB may be secreted through the apparatus and docked on the end of the needle complex before CopN plugs the needle apparatus. Considering the fact that recent work has suggested that Cpn0803 may be a chaperone protein given its biophysical properties and putative interactions, it is not surprising that CopB failed to interact with Cpn0803 [29, 30]. The interaction between CopB and the needle filament protein, CdsF, in this report is a novel observation not previously reported in the literature. It has been reported that the translocators are recruited to the tip of the needle complex either upon detecting host cell contact or under secretion conditions. Once the translocators are inserted into the host cell membrane, the filament protein must anchor to the host cell via the translocator proteins, which are now imbedded in the host membrane. This result is consistent with the role of the hydrophobic translocator proteins in other T3SS since the needle protein must interact with the translocator proteins on the host cell to facilitate translocation of effector proteins [31, 32].

Interactions between class II chaperones and translocator proteins have been documented in *Chlamydiae spp.* previously. Initial identification of the LcrH_1 and CopB interaction, from *C. trachomatis*, was performed by Fields et al. (2005) [10, 21]. Using a GST pulldown, we demonstrated that the N-terminus of CopB_1–255_ interacts with LcrH_1, which is in keeping with LcrH_1 orthologs interacting within the N-terminus of translocator proteins [28, 33]. An additional truncation series showed that the removal of the hydrophobic amino acids from 180 to 200 eliminated the interaction of LcrH_1 and CopB despite the presence of the PxLxxP motif within the CopB_1–180_ construct. The PxLxxP motif is conserved in members of the *Chlamydiaceae* family, despite the low amino acid sequence identity, suggestive of an important role for the chaperone binding motif (Table 1). An alanine walkthrough of the conserved amino acids in the PxLxxP motif disrupted the interaction between CopB_1–200_ and LcrH_1. Our data indicates that the interaction between CopB and LcrH_1 is dependent on both the PxLxxP motif and the CopB_180–200_ domains.

**Table 1:** Comparison of putative chaperone binding domains between *Chlamydiaceae* family members and other T3SS containing Gram-negative bacteria

	P1		P3			P6	Percent identity
CopB (*C. pneumoniae*)	P	E	L	P	K	P	100 %
CT578 (*C. trachomatis serovar D*)	P	G	L	P	K	P	52 %
SseC like family protein (*C. psittaci*)	P	D	L	P	K	P	53 %
TC_0867 (*C. muridarum*)	P	G	L	P	K	P	50 %
CPE1_0913 (*C. pecorum*)	P	E	L	T	P	P	53 %
CAB923 (*C. abortus S26/3*)	P	D	L	P	K	P	54 %
PopB (*Y. enterocolitica*)	P	A	L	G	R	P	18 %
IpaB (*S. dyseteriae*)	P	E	L	K	A	P	17 %

Putative chaperone binding domains were identified within the N-terminal regions of orthologous proteins to CopB from *C. pneumoniae.* P1, P3, P6, represent positions 1, 3, and 6, respectively of the PxLxxP motif. Percent identity refers to amino acid sequence identity comparing full length CopB to full length sequences of orthologous proteins

The co-crystal structure of class II chaperones (LcrH_1 orthologs) with the translocator PxLxxP domain confirms the interaction between the two proteins [28]. Based on this interaction we hypothesized that a cognate peptide of CopB containing the PxLxxP peptide sequence could disrupt the translocator-chaperone interaction. We therefore tested a peptide containing the 10 amino acids encompassing the PxLxxP domain plus a cell penetrating peptide sequence to test this hypothesis. Since the PxLxxP motif is conserved between CopB and CopD, we pre-incubated LcrH_1 with the cognate peptide then added either CopB or CopD fragments to the GST pull down and showed that the peptide blocked the interaction between LcrH_1, and CopB and CopD. Since the cell penetrating peptide sequence allows proteins to enter cells, we hypothesized that this cognate peptide would block chlamydial infection and intracellular replication [34, 35]. After pre-treating *C. pneumoniae* with this peptide, we observed a significant reduction of 90 %, compared to control infection, with vehicle alone. Since it is currently not possible to create genetic knockouts in *Chlamydia*, peptide mimetics could be used to create functional knockouts to study the resultant phenotype. The peptides ability to significantly reduce infection reinforces the importance of the chaperone binding motif for the chaperone-translocator interaction and may represent a novel target for therapeutic intervention using peptide mimetics.

## Conclusions

Antibodies to the translocator proteins in orthologous secretion systems have been shown to inhibit infection, suggesting that the translocator proteins play an essential role during infection [36–40]. Using antibodies raised to an N-terminal epitope of CopB, we demonstrated that anti-CopB antibodies inhibited *C. pneumoniae* infection by 98 %. Inhibition of infection by anti-CopB antibodies indicates that CopB is surface exposed at some time during infection and play an essential role in infection. Given the fact that CopB is surface exposed during the initial phase of chlamydial infection, the translocator proteins may represent a novel class of antigens for use in vaccination strategies to prevent chlamydial infections.
